# Classification of maxillectomy in edentulous arch defects, algorithm, concept, and proposal classifications: A review

**DOI:** 10.1002/cre2.708

**Published:** 2023-01-04

**Authors:** Hatem Alqarni, Mohammed Alfaifi, Walaa Magdy Ahmed, Rania Almutairi, Mathew T. Kattadiyil

**Affiliations:** ^1^ Department of Restorative and Prosthetic Dental Sciences, College of Dentistry King Saud Bin Abdulaziz University for Health Sciences Riyadh Saudi Arabia; ^2^ King Abdullah International Medical Research Center Riyadh Saudi Arabia; ^3^ Department of Prosthtic Dental Sciences, King Khalid University College of Dentistry, Abha, Saudi Arabia; Fellow in Advanced Digital Prosthodontics and Implant Dentistry, Department of Prosthodontics Loma Linda University School of Dentistry Loma Linda California USA; ^4^ Department of Restorative Dentistry, Faculty of Dentistry King Abdulaziz University Jeddah Saudi Arabia; ^5^ Prosthodontic Resident King Saud University Riyadh Saudi Arabia; ^6^ Advanced Education Program in Prosthodontics Loma Linda University School of Dentistry Loma Linda California USA

**Keywords:** Armany, defect, maxillectomy, oncology

## Abstract

**Objectives:**

Aramany's classification of postsurgical maxillectomy defects was introduced for partially edentulous situations, and has been widely used for education and effective communication among practitioners. Numerous classifications of maxillofacial defects, based on surgical procedure, resultant defects, or prosthodontist's perspective after rehabilitation, exist in the literature. However, no single classification has incorporated all these factors. The purpose of this review was to highlight the classification systems and describe a pragmatic classification series for edentulous maxillary arch defects (maxillectomy) by applying the Aramany classification criteria, to enhance treatment outcomes and communication among practitioners.

**Material and Methods:**

An electronic search of the literature published in English was conducted using the PubMed/MEDLINE and Google Scholar database. Keywords used were “maxillectomy classification” AND “surgical resection,” “maxillectomy classification” AND “complete edentulous.” In addition, a manual search was also performed followed the same criteria in the following journals: Journal of Prosthetic Dentistry and Journal of Prosthodontics.

**Results:**

Several classification systems for partial dentition were found in terms of size, location, dentition, and extension of the defect (isolated or communication defects). The findings revealed a variety of maxillectomy defect classifications for partially dentate, considering surgical factors and rehabilitation. However, no study or classification system exist for the edentulous arch defects.

**Conclusions:**

Different classification systems for maxillectomy defects exist in the literature, only for partially dentate patients. To the authors best knowledge, no classification system for completely edentulous maxillary arch defects have been proposed till date. A simple classification system with clear characteristics for edentulous maxillectomy dental arch defects has been proposed. This classification was modeled after Aramany classification for easier memorization and application.

## Clinical implication

This article reviews maxillectomy defect classifications in the literature. A classification for maxillectomy defects of the edentulous arch was proposed using the concepts of the Aramany classification.

## 1 INTRODUCTION

Maxillectomy or maxillary resection is defined as the surgical removal of part or all of the maxilla (The glossary of prosthodontic terms, 2017). The surgeon and the reconstructive team make individualized decisions based on the size and extent of the defects. Consequently, due to the complexity and three‐dimensional architecture of the maxilla, there have been several attempts to achieve a unified classification system of midface post‐ablative defects. Seventeen different classifications have been proposed in the past half‐century (The glossary of prosthodontic terms, 2017). However, none of the existing classifications concisely covers all possible defects, which is essential for effective communication among practitioner's to develop an appropriate treatment plan (Akinmoladun et al., 2013; Aramany, 1978; Bidra et al., 2012; Brown & Shaw, 2010; Brown et al., 2000; Carrillo et al., 2005; Cordeiro & Santamaria, 2000a, 2000b; Costa et al., 2015; Davison et al., 1998; Durrani et al., 2013; Futran & Mendez, 2006; Ohngren, 1975; Okay et al., 2001; Rodriguez et al., 2007; Shrime & Gilbert, 2009; Spiro et al., 1997; Triana et al., 2000; Umino et al., 1998; Wells & Luce, 1995; Yamamoto et al., 2004).

Even with the advent of numerous classification systems, there still remains a confusion regarding the use of terminologies such as limited, partial, subtotal, and total. Bidra et al. suggested six criteria for the universal assessment of these existing classifications and concluded that no one system has succeeded in including all the relevant criteria (Bidra et al., 2012).

Two grouped categories of defect classifications in the literature are based on the extension of surgical resection and the remaining teeth, or from the prosthodontist's and/or head and neck surgeon's perspective after completion of surgical reconstruction of the defect (Aramany, 1978; Brown et al., 2000; Cordeiro & Santamaria, 2000a; Davison et al., 1998; Spiro et al., 1997; Triana et al., 2000; Umino et al., 1998; Wells & Luce, 1995). The earliest and simplest classification of maxillary diseases was given by Ohngren in 1933 (Ohngren, 1975). Since Ohngren did not believe in radical resection of maxillary tumors, his classification system was based on establishing resectability criteria and did not consider the postsurgical defect (Akinmoladun et al., 2013; Ohngren, 1975).

Aramany in 1987 was the first to describe the surgical defect of partially dentate maxilla (Aramany, 1978). His classification was based on the frequency of occurrence of defects in a cohort of 123 patients (Aramany, 1978; Bidra et al., 2012; Brown & Shaw, 2010; Brown et al., 2000; Carrillo et al., 2005; Cordeiro & Santamaria, 2000a; Davison et al., 1998; Futran & Mendez, 2006; Okay et al., 2001; Rodriguez et al., 2007; Shrime & Gilbert, 2009; Spiro et al., 1997; Triana et al., 2000; Umino et al., 1998; Wells & Luce, 1995; Yamamoto et al., 2004). The maxillectomy classification by Aramany was put forward for partially edentulous patients which grouped particular combinations of teeth and surgical defects; and made it an effective tool for better communication and development of appropriate framework designs for obturator prostheses (Aramany, 1978). In the Aramany era of classification systems, the defects were characterized simply based on anatomic landmarks of the defects and lacked appropriate algorithms for reconstruction (Aramany, 1978). However, the current literature has focused more on reconstruction options, for specific defects.

The purpose of this review was to map all maxillary arch defect classification systems available in the literature and describe a pragmatic classification series for edentulous maxillary arches with defects by applying the Aramany classification criteria to enhance treatment outcomes and communication among practitioners.

## 2 MATERIALS AND METHODS

### 2.1 Protocol and registration

This review was conducted according to the Preferred Reporting Items for Systematic Reviews and Meta‐Analyses (PRISMA) protocol. The review protocol was registered at the Prospectively Registered Systematic Reviews (PROSPERO) platform under (370285).

### 2.2 Focused question and search strategy

The focused question was determined according to the 2009 PICO strategy (Furlan et al., 2009). (1) Population: maxillary arch OR complete edentulous OR partial edentulous OR dentate; (2) Intervention: surgical resection OR maxillectomy OR maxillary arch resection; (3) Comparison: N/A. (4) Outcome: classification OR category OR types. The focused questions of the present review was “Among the available studies on maxillary arch defect classifications, what factors are the classification systems based on? Is the available maxillectomy classification system applicable to edentulous maxillary arches?

### 2.3 Selection criteria

Inclusion criteria

1‐ In vivo studies that classified maxillary arch defect, resection, or maxillectomy.
2‐ Patients with dentation, partial dentation, or edentulous maxillary arch.
3‐ Any of the following outcomes were evaluated; classification, categorization, and/or types.

Exclusion criteria

1‐ In vivo studies which classified mandibular arch defect, resection or mandibulectomy.
2‐ Animal studies.

### 2.4 Search methods

Two independent authors (H. Q. and WMA) systematically searched the indexed literature in January 2022, and updated it in August 2022. An electronic search of the literature related to this subject between 1978 and 2022 was performed, with specified classifications of dentoalveolar defects in Cochrane Library, PubMed/MEDLINE and Google Scholar using the search terms “maxillectomy classification” AND “surgical resection”, “maxillectomy classification” AND “complete dentulous.” Combinations of medical subject heading terms (MeSH) and non‐MeSH terms, along with Boolean operators, were utilized to perform the search. Relevant literature was also searched through Open Grey until January 2022. A manual search of the available literature was also performed without any language or publication restrictions. The following journals were manually checked: Journal of Prosthetic Dentistry, Journal of Prosthodontics, and Advanced Journal of Prosthodontics.

### 2.5 Study selection and data extraction

EndNote citation manager was used to import all the articles collected from the three databases. Subsequently, duplications were removed. Titles and abstracts were filtered (by H. Q. and WMA) according to the inclusion and exclusion criteria. Any disagreements between reviewers were resolved through discussion. Full‐text screenings were performed, and data related to classification of maxillary arch defects were obtained.

### 2.6 Quality assessment

Interobserver calibration was evaluated using Cohen's Kappa, and the chosen cutoff point was 80%. GRADE criteria were used to provide a framework for quality assessment of the selected studies.^25^ The quality levels ranged between high (H) and moderate (M), Low (L), and very low (VL). Quality reflects the confidence that the estimate of the effect was correct. GRADE separates the process of quality assessment of evidence from that of formulating recommendations (Balshem et al., 2011).

## 3 RESULTS

Electronic searches using Cochrane, PubMed/MEDLINE, and Google Scholar along with manual searches, were performed. A total of 570 articles were screened and 17 published research were selected for data extraction. The characteristics of each classification are summarized in Table 1 (Bidra et al., 2012).

**Table 1:** Details of all the classifications included in this review

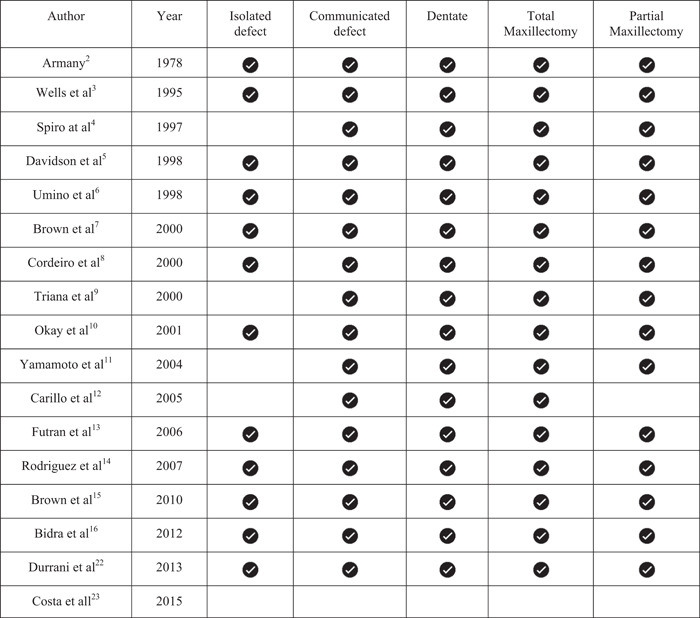

## 4 DISCUSSION

Defect extension in the maxillary arch has been described in multiple planes as involvement of different regions of the maxilla (Aramany, 1978; Bidra et al., 2012; Brown & Shaw, 2010; Okay et al., 2001). It is important to recognize the location of defect, as treatment modality varies significantly between the anterior and posterior regions of the maxilla (Aramany, 1978; Bidra et al., 2012; Okay et al., 2001). Anterior region defects result in lip incompetency, drooling, speech difficulty, collapse and deformity of nose, trismus, and formation of scar tissues, all of which have to be factored in the prosthesis design (Bidra et al., 2012). Arch discrepancy from bilateral defects involving maxillae would require reverse articulation of denture teeth arrangement (Bidra et al., 2012). Treatment planning for posterior defect rehabilitation may be affected by the resection of the soft palate, which could result in insufficient/inadequate velopharyngeal closure. This would require fabrication of a palatopharyngeal obturator to achieve adequate soft palate function and velopharyngeal competency (Bidra et al., 2012).

The obturator extends to the inferior conchae or even further distally to the posterior pharyngeal wall in cases of bilateral posterior defects (Class V) and soft palate resections. This can lead to loss of retention and stability due to increase in size and weight of the obturator; and also cause leakage of fluids and air if there are no existing teeth or endosseous implants (Bidra et al., 2012). The placement of endosseous dental implants in the residual alveolar ridge of the maxilla can improve retention and stability of the obturator (Aramany, 1978; Bidra et al., 2012; Okay et al., 2001). Only three of the existing 17 classifications were based on prosthetic considerations. These are the classifications by Aramany (1978); Okay et al. (2001); and Rodriguez et al. (2007).

Aramany classification remained the standard for almost two decades. However, his classification was devised purely from a prosthodontic perspective, and mainly discussed the palate and alveolar ridge. It did not address the involvement of contiguous structures such as nose, cheek, and outer skin, orbital contents, zygoma, and pterygoid plates; thus, was not an effective tool for describing surgical defects (Bidra et al., 2012; Shrime & Gilbert, 2009).

Spiro et al. (1997) described the difficulty with maxillary defect classification and proposed a classification system based on retrospective evaluation of 442 maxillectomies and orofacial resections performed over a period of 9 years. They proposed three types of procedures in this classification scheme: Class I, limited maxillectomy with involvement of one wall; Class II, subtotal maxillectomy with involvement of at least two walls including the palate, subdivided further into anterior, inferior medial, and lateral types; Class III, total maxillectomy which was complete resection of maxilla with or without orbital involvement (Spiro et al., 1997). Brown et al. proposed the first classification since Aramany's, which focused on both surgical and prosthodontic approaches toward a classification of palatal defects (Bidra et al., 2012; Brown & Shaw, 2010; Shrime & Gilbert, 2009). After analyzing 487 patients, they divided the maxillary and midface defects based on the vertical (Classes 1–4) and horizontal (classes a–c) components of tissue defects (Bidra et al., 2012; Brown & Shaw, 2010; Shrime & Gilbert, 2009).

The surgical components (vertical) were as follows: Class 1, maxillectomy not causing oronasal fistula; Class 2, maxillectomy not involving the orbit; Class 3, maxillectomy involving the orbital adnexae with orbital retention; Class 4, maxillectomy with orbital enucleation or exenteration; Class 5, orbitomaxillary defect; and Class 6, nasomaxillary defect.

The dental components (horizontal) were: a, palatal defect only; b, less than or equal to half of the bilateral maxilla; c: less than or equal to half of the unilateral maxilla; and d, greater than half of the maxillectomy.

Brown's intention was to provide a framework for prosthodontic rehabilitation, predict future prognosis, and guide the surgeons in reconstructing the defect. In 2010, this system was revised to account for the orbitomaxillary region as a Class V defect and nasomaxillary defects as Class VI, as well as the reconstructive approach, according to the given defect (Bidra et al., 2012; Brown & Shaw, 2010; Shrime & Gilbert, 2009).

However, this system is comprehensive and too complicated to use. The classification given by Brown et al. failed to describe the anterior‐posterior extent and skull base defect (Bidra et al., 2012; Shrime & Gilbert, 2009). In addition, it did not mention the amount of skin loss and status of the palate and dentition (Bidra et al., 2012; Shrime & Gilbert, 2009).

ln the same year, Cordeiro and Santamarial expanded the Spiro classification, and proposed a classification system and an algorithm for reconstruction of these defects by measuring the surface area and using a wide variety of flaps (Cordeiro & Santamaria, 2000b). Their classification was as follows:

Type 1, limited maxillectomy, resection of one or two walls of the maxilla with preservation of the palate.

Type 2, sub‐total maxillectomy, resection of the maxillary arch, palate, anterior and lateral walls, and five out of six walls of maxilla with preservation of the orbital floor; Type 3, total maxillectomy, resection of all six walls of maxilla with preservation of orbital contents. This type was further divided into two parts: Type 3a: total maxillectomy with preserved orbital contents, type 3b: total maxillectomy with orbital exenteration; Type 4: orbito‐maxillectomy, orbital exenteration with resection of five walls of maxilla, preserving the palate.

Reconstruction Algorithm:

Type 1 Defect: Reconstruction with free non‐vascularized bone may be required to replace bone in the critical area. Further obliteration can be performed using radial forearm fasciocutaneous flap (RFFF).

Type 2 Defect. RFFF can be used to reconstruct the missing palate. An osseo‐facio‐cutaneous RFFF can be used to reconstruct anterior maxilla, which will also provide good lip support. Type 3a Defect: Free nonvascularized bone can be used to reconstruct the orbital floor, and remaining defect can be closed using rectus abdominus or temporalis flap.

Type 3b Defect: Reconstruction can be performed using rectus abdominus flap with skin paddles to reconstruct the palate, nasal wall, or facial skin.

Type 4 Defect: Reconstruction can be performed using a rectus abdominus flap with or without skin paddles.

In 2000, Triana et al. (2000) classified and divided the defects into three classes based on vertical extension and the affected area of the palate. They designated their classification as (1) inferior partial maxillectomy, while the subdivision was based on the extent of loss of palate; and (2) total maxillectomy, subdivided based on orbital exenteration, and amount of loss of malar bone and zygomatic arch (Triana et al., 2000). This system was not comprehensive and failed to facilitate easy communication (Akinmoladun et al., 2013; Bidra et al., 2012; Cordeiro & Santamaria, 2000b; Ohngren, 1975; Shrime & Gilbert, 2009; Triana et al., 2000).

In the following year, Okay et al. (2001) proposed to organize and define the nature of prosthetic decision‐making and patient satisfaction. Moreover, they were the first to directly consider the status of zygomatic arch and orbital floor (Okay et al., 2001). This classification system was based on a retrospective review of 47 consecutive maxillectomy defects, and divided them into three major classes and two subclasses (Okay et al., 2001).

Class I a: Defects that involve any portion of the hard palate, but not the tooth‐bearing alveolus.

Class I b: Defects that involve any portion of the maxillary alveolus and dentition posterior to the canines or involve the pre‐maxilla with preservation of both canines.

Class II: Defects that involve less than half of the hard palate of tooth‐bearing area and include only one canine.

Class III: Defects involving resection of both canines or more than half of the hard palate; Class III f includes defects that involve the inferior orbital rim; and Class III z, includes defects that involve the body of the zygomatic bone.

For reconstructive outcomes, Okay et al. were the only ones to describe the outcomes after rehabilitation for each defect (Okay et al., 2001). However, this classification was also very complicated and failed to address defects involving contiguous contents such as orbital contents, soft palate, facial skin, and base of skull (Bidra et al., 2012; Ohngren, 1975; Okay et al., 2001; Shrime & Gilbert, 2009). The Okay classification system mainly considered dental and alveolar restoration for obturator stability and retention (Bidra et al., 2012; Ohngren, 1975; Okay et al., 2001; Shrime & Gilbert, 2009).

Umino et al. (1998) measured the speech intelligibility of 54 patients with or without a prosthesis after maxillectomy. They concluded that oronasal communication played a major role in speech intelligibility without a prosthesis; and developed a classification system to help predict the grade of post‐maxillectomy speech disorder following surgery (Umino et al., 1998). They designated their classification based on location of the defects, either in the hard palate (Class I) or soft palate (Class II), and sub‐classified these based on connections with the antral and nasal cavities (Umino et al., 1998).

Literature has classified defects based on their location, which can be isolated, with or without oroantral communication, unilateral or bilateral (Bidra et al., 2012; Brown & Shaw, 2010; Brown et al., 2000; Rodriguez et al., 2007). In partially dentate situations, due to availability of surface area and abutments that can provide adequate retention and stability for the obturator, management of isolated or unilateral defects (Class II–III, VI) can be surgically or prosthetically achieved based on the size and location of the defect (Bidra et al., 2012). However, in edentulous maxillary arch bilateral defects (Class I, II, IV, V, and VII) involving both maxillae, placement of endosseous implants to provide support and stability of the prosthesis is indicated (Bidra et al., 2012; Okay et al., 2001). Other authors have reported considerations for prosthodontic rehabilitation of unilateral and bilateral defects in partially dentate patients (Aramany, 1978; Bidra et al., 2012; Okay et al., 2001).

Durrani et al. (2013) proposed a maxillectomy classification based on the clinician's guidance of the reconstructive and rehabilitation options. Type 1: alveolectomy, the surgical defect that involves only alveolar bone with, no oronasal or oroantral fistulas, which could be covered by the denture; Type 2: subtotal maxillectomy, the surgical defect that involves oronasal or oroantral fistula without disturbing the orbital walls, where obturator or local flap can be used; Type 3, total maxillectomy, the surgical defect involves complete removal of maxilla and orbital floor without involving the orbital contents. An obturator that extends to the orbit or regional flap can be used. Type 4: radical maxillectomy, when the resection involves removal of the orbital contents alone with the maxilla and orbital floor. A prosthetic appliance and eyeball alone with skin graft can be used; and Type 5: composite maxillectomy, when facial skin, soft palate, and/or other parts of the oral cavity are resected with the maxilla. Durrani et al. classification can be subdivided into unilateral and bilateral defects (Durrani et al., 2013).

On the other hand, Costa et al. (2015) classified maxillary arch defects into: 1) Type 1 defects or limited maxillectomy, which includes resection of one or three walls of maxilla with or without palate; 2) Type II defects or subtotal or infrastructural maxillectomy, which includes resection of five walls of the maxilla; 3) Type III defects or total maxillectomy, which includes resection of six walls of the maxilla; and 4) Type IV defects or orbital or suprastructural maxillectomy, which includes resection of five walls of the maxilla along with the orbital contents, with preservation of palate and maxillary arch (Costa et al., 2015).

## 5 PROPOSED UNIVERSAL CLASSIFICATION

**Figure 1:**
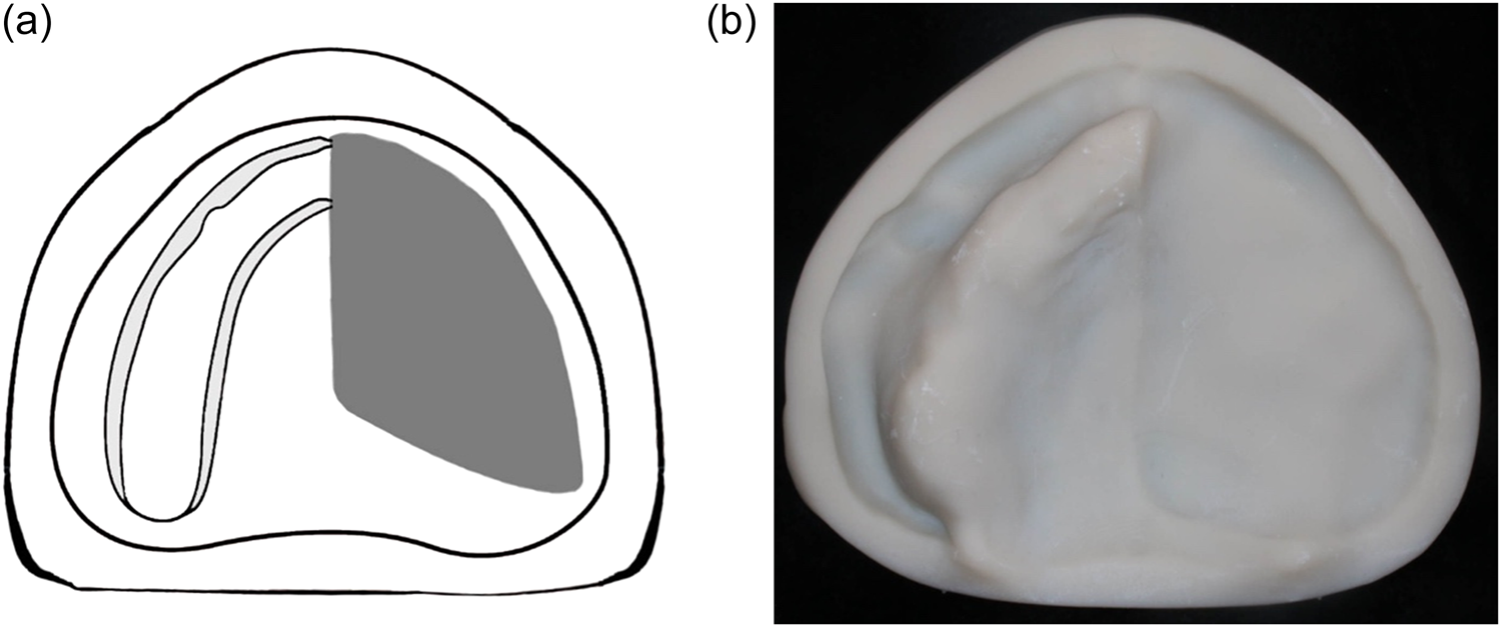
Classification for edentulous dental arches with maxillectomy defects: (a) Schematic diagram designed according to the proposed classification. (b) Occlusal view of the printed cast showing the defect. (a and b). Class I, midline resection.

To discuss edentulous arch defects in maxillectomy patients in a simple and effective manner, a universal classification is shown in Table 2. The horizontal components of the proposed classification is divided into seven groups based on the relationship between defect area and remaining edentulous area (Figure 1). The sequence of the Aramany classification for partially edentulous patients was followed (Aramany, 1978).

**Table 2:** Proposed treatment options for individual classification defect

D Superstructural or Orbitomaxillectomy	C Total Maxillectomy Total	B Infrastructural	A Limited	Resection
Myocutaneous rectus abdominis (McRA) Myocutaneous latissimus dorsi (McLD)	Myocutaneous rectus abdominis (McRA) Myocutaneous latissimus dorsi (McLD)	OmIC‐osteomuscular Iliac crest FRF‐faciocutaneous radial forearm	X	0
Myocutaneous latissimus dorsi (McLD)	OmIC‐osteomuscular Iliac crest OmFe‐osteomnuscular fibula Myocutaneous rectus abdominis (McRA) OmcF‐osteomyocutaneous fibula	FRF‐faciocutaneous radial forearm	Obturator Prosthesis	1 Midline Midline
X	X	**N/A**	Obturator Prosthesis	2 Unilateral
X	X	X	Obturator Prosthesis	3 Central
X	**N/A**	OFRF‐osteofasciocutaneous radial forearm OmIC‐osteomuscular Ilic crest OmcF‐osteomyocutaneous fibula	**N/A**	4 Bilateral
X	X	N/A	FRF‐faciocutaneous radial forearm OFRF‐osteofasciocutaneous radial forearm	5 Posterior
X	X	N/A	OmIC‐osteomuscular Iliac crest OFRF‐osteofasciocutaneous radial forearm	6 Anterior
X	X	N/A	FRF‐faciocutaneous radial forearm OFRF‐osteofasciocutaneous radial forearm	7 Middle

Description: Classification for edentulous dental arches with maxillectomy defects: Zero mean does not belong to any of these criteria or classification, Class I, midline resection, Class II, unilateral resection, Class III, central resection of the hard palate, Class IV, bilateral anterior‐posterior resection extended beyond the incisive papillae, Class V, posterior resection, Class VI, anterior resection, and Class VII, middle resection of the remaining residual alveolar ridge bilaterally.

Class I: The defect is located along the midline of the maxillary arch, which involves the incisive papilla (Figure 1a,b).

**Figure 2:**
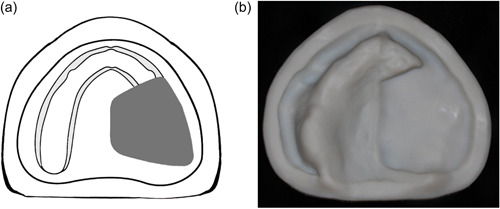
(a) and (b) Class II, unilateral resection.

Class II: Unilateral defect with intact anterior, and one side of the residual alveolar ridge on the contralateral side (Figure 2a,b).

**Figure 3:**
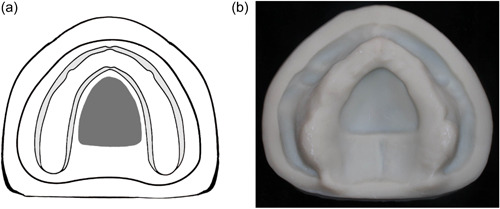
(a) and (b) Class III, central resection of the hard palate.

Class III: The defect occurs in the middle portion of the hard palate with intact remaining residual alveolar ridge (Figure 3a,b).

**Figure 4:**
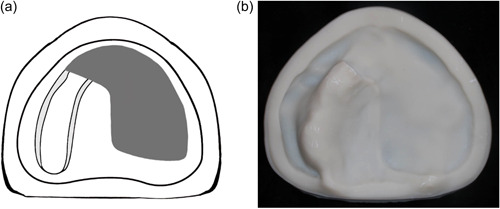
(a) and (b) Class IV, bilateral anterior‐posterior resection extended beyond the incisive papillae.

Class IV: The defect crosses the midline and involves both sides of the maxilla (Figure 4a,b).

**Figure 5:**
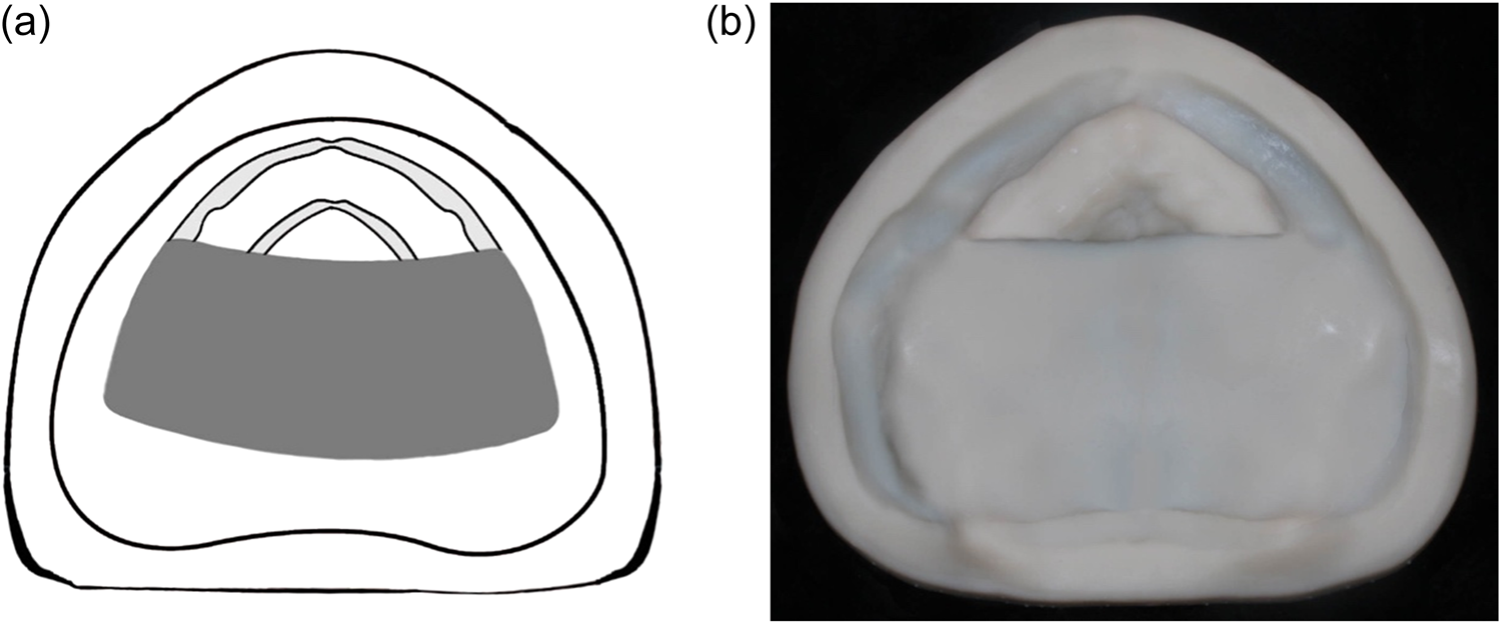
(a) and (b) Class V, posterior resection.

Class V: Bilateral defect posterior to the remaining residual alveolar ridge (Figure 5a,b).

**Figure 6:**
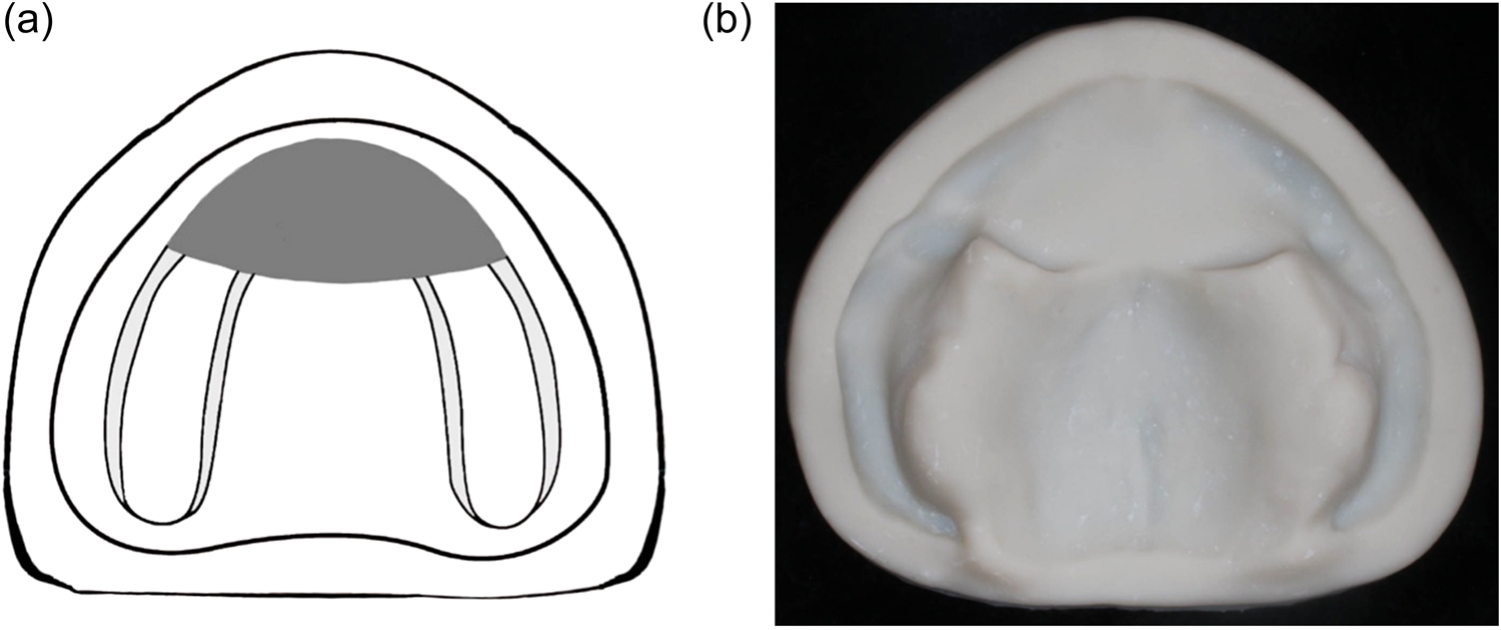
(a) and (b) Class VI, anterior resection.

Class VI: Defects occur in the anterior region of the maxillary alveolar ridge, preserving the posterior portion bilaterally. This is a common presentation of congenital defects and defects caused by trauma (Figure 6a,b).

**Figure 7:**
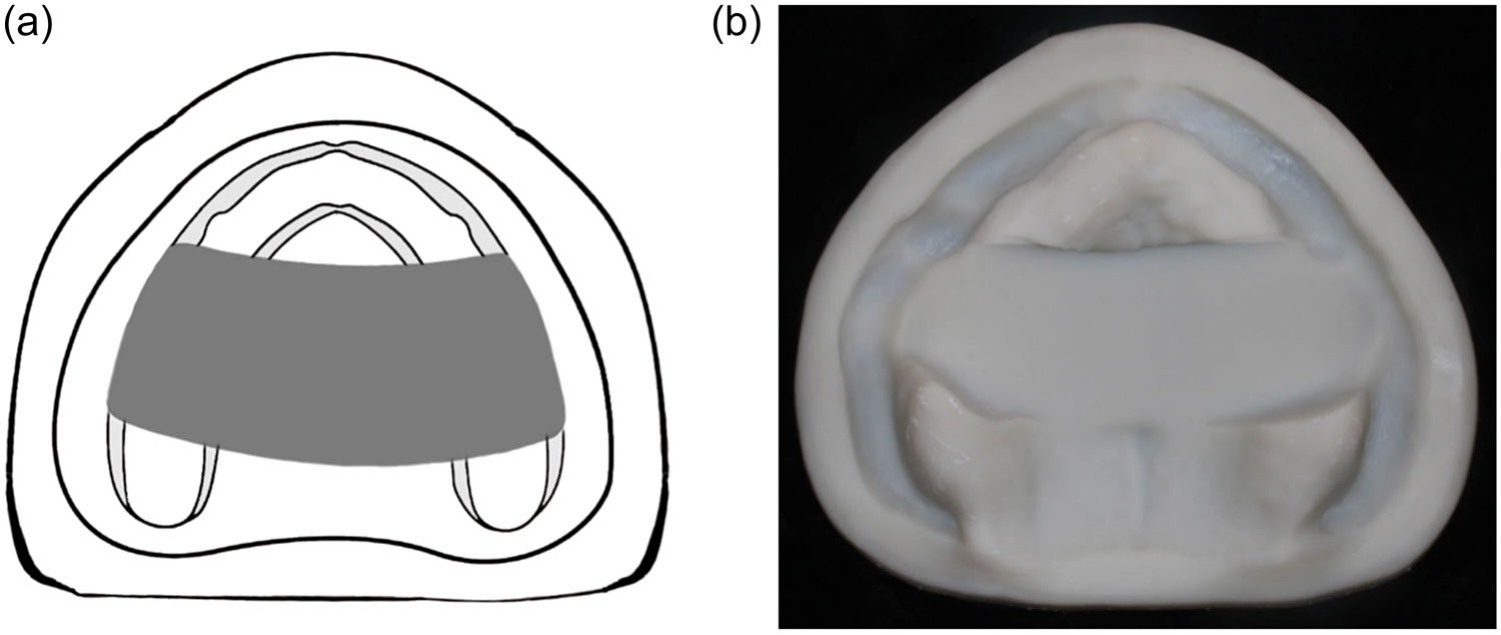
(a) and (b) Class VII, middle resection of the remaining residual alveolar ridge bilaterally.

Class VII: The defect cross the middle region of the alveolar ridge and involves both sides of the maxilla. A part of the residual alveolar ridge remains, which lies posterior and anterior to the defect (Figure 7a,b).

The vertical component of the proposed classification was divided into four categories by alphabetical letters: A, B, C, and D.

A‐ Category: Limited to maxilla, including resection of one or three walls of the maxilla with or without comprising the palate. It can be combined with horizontal components of Classes 1, 2, 3, 4, 5, 6, and 7.
B‐ Category: Infrastructural including resection of the lower five maxillary walls, including maxillary arch, palate, anterior, posterior, medial, and lateral walls without the orbital floor. It can be combined with horizontal components of Classes 1, 2, 4, 5, 6, and 7.
C‐ Total maxillectomy includes resection of all six walls of the maxilla, with or without the orbital content. This can be combined with horizontal components 1 and 4.
D‐ Orbitomaxillectomy or suprastructural resection includes resection of the upper five walls of maxilla with orbital contents, and without palate or maxillary arch. It cannot be combined with any horizontal component. It can be managed using selected free flaps, such as myocutaneous rectus abdominis or myocutaneous latissimus dorsi.

This proposed classification provides an accurate description of maxillectomy defect/maxillary resection for edentulous patients in horizontal and vertical perspectives, and allows a realistic comparison of pretreatment and posttreatment outcomes of surgical reconstruction and/or prosthodontic rehabilitation of maxillary defects. The authors believe that the development of a systematic classification of maxillectomy defects for edentulous patients has value for students, teachers, and practitioners. The presence of several classification systems in the literature demonstrates a lack of consensus and reveals the challenges involved in classifying maxillectomy defects in partially edentulous patients and lack of information and standardized classification for edentulous patients. This classification can also help standardize categorization for future research on maxillectomy defects.

## 6 CONCLUSIONS

Different classification systems for maxillectomy defect exist only for partially dentate patients. To the author's best knowledge, no classification system exists for edentulous maxillary defects. A simple classification system with clear characteristics for edentulous maxillectomy dental arch defects has been proposed. This classification was modeled after the Aramany classification, which makes it easier to memorize and apply.

## AUTHOR CONTRIBUTIONS

Hatem Alqarni contributed to the design of the study, search, and selection, and drafted the manuscript. Hatem Alqarni, Mohammed Alfaifi, Mathew Kattadiyil contributed to the study, and Hatem Alqarni, Rana Almutairi, and Walaa Magdy contributed to the analysis and interpretation and critically revised the manuscript. Hatem Alqarni, Mohammed Alfaifi, Walaa Magdy Rana Almutairi, and Mathew Kattadiyil contributed to the conceptualization and design of the study, search and selection, analysis and interpretation, and critically revised the manuscript. All the authors gave final approval and agreed to be accountable for all aspects of the work, ensuring integrity and accuracy.

## Data Availability

NA Comments to Payment Admin.
